# Impact of diaphragm paralysis and its surgical interventions on outcomes after the staged Fontan procedure

**DOI:** 10.1093/icvts/ivaf072

**Published:** 2025-03-19

**Authors:** Ruxandra Dumitru, Muneaki Matsubara, Thibault Schaeffer, Takuya Osawa, Jonas Palm, Carolin Niedermaier, Nicole Piber, Paul Philipp Heinisch, Bettina Ruf, Alfred Hager, Peter Ewert, Jürgen Hörer, Masamichi Ono

**Affiliations:** Department of Congenital and Pediatric Heart Surgery, German Heart Center Munich, Technische Universität München, Munich, Germany; Division of Congenital and Pediatric Heart Surgery, University Hospital of Munich, Ludwig-Maximilians-Universität München, Munich, Germany; Europäisches Kinderherzzentrum München, Munich, Germany; Department of Congenital and Pediatric Heart Surgery, German Heart Center Munich, Technische Universität München, Munich, Germany; Division of Congenital and Pediatric Heart Surgery, University Hospital of Munich, Ludwig-Maximilians-Universität München, Munich, Germany; Europäisches Kinderherzzentrum München, Munich, Germany; Department of Congenital and Pediatric Heart Surgery, German Heart Center Munich, Technische Universität München, Munich, Germany; Division of Congenital and Pediatric Heart Surgery, University Hospital of Munich, Ludwig-Maximilians-Universität München, Munich, Germany; Europäisches Kinderherzzentrum München, Munich, Germany; Department of Congenital and Pediatric Heart Surgery, German Heart Center Munich, Technische Universität München, Munich, Germany; Division of Congenital and Pediatric Heart Surgery, University Hospital of Munich, Ludwig-Maximilians-Universität München, Munich, Germany; Europäisches Kinderherzzentrum München, Munich, Germany; Department of Congenital Heart Disease and Pediatric Cardiology, German Heart Center Munich, University Hospital of Technische Universität München, Munich, Germany; Department of Congenital and Pediatric Heart Surgery, German Heart Center Munich, Technische Universität München, Munich, Germany; Division of Congenital and Pediatric Heart Surgery, University Hospital of Munich, Ludwig-Maximilians-Universität München, Munich, Germany; Europäisches Kinderherzzentrum München, Munich, Germany; Department of Cardiovascular Surgery, German Heart Center Munich, Technische Universität München, Munich, Germany; Department of Congenital and Pediatric Heart Surgery, German Heart Center Munich, Technische Universität München, Munich, Germany; Division of Congenital and Pediatric Heart Surgery, University Hospital of Munich, Ludwig-Maximilians-Universität München, Munich, Germany; Europäisches Kinderherzzentrum München, Munich, Germany; Department of Congenital Heart Disease and Pediatric Cardiology, German Heart Center Munich, University Hospital of Technische Universität München, Munich, Germany; Department of Congenital Heart Disease and Pediatric Cardiology, German Heart Center Munich, University Hospital of Technische Universität München, Munich, Germany; Department of Congenital Heart Disease and Pediatric Cardiology, German Heart Center Munich, University Hospital of Technische Universität München, Munich, Germany; Department of Congenital and Pediatric Heart Surgery, German Heart Center Munich, Technische Universität München, Munich, Germany; Division of Congenital and Pediatric Heart Surgery, University Hospital of Munich, Ludwig-Maximilians-Universität München, Munich, Germany; Europäisches Kinderherzzentrum München, Munich, Germany; Department of Congenital and Pediatric Heart Surgery, German Heart Center Munich, Technische Universität München, Munich, Germany; Division of Congenital and Pediatric Heart Surgery, University Hospital of Munich, Ludwig-Maximilians-Universität München, Munich, Germany; Europäisches Kinderherzzentrum München, Munich, Germany

**Keywords:** Fontan, single ventricle, diaphragm paralysis, diaphragm plication, protein-losing enteropathy

## Abstract

**OBJECTIVES:**

This study aimed to investigate the incidence of diaphragm paralysis and its impact on outcomes after the Fontan procedure in patients with single ventricles.

**METHODS:**

Patients undergoing staged Fontan palliation between 1994 and 2023 were reviewed. Patients who had diaphragm paralysis were identified, and the impact of diaphragm paralysis on outcomes after Fontan completion was evaluated.

**RESULTS:**

Among 601 patients who underwent staged Fontan completion during the study period, diaphragm paralysis was observed in 79 patients (13.1%) before Fontan (33 after stage I palliation and 46 after Glenn) and in 32 patients (5.3%) after the Fontan. Among 111 patients with diaphragm paralysis, 77 had spontaneous recovery, 13 recovered after plication and 21 remained without recovery. Patients with diaphragm paralysis before the Fontan demonstrated higher pulmonary arterial pressure (median 10 vs 9 mmHg, *P* = 0.045) and lower pulmonary artery symmetry index (median 0.54 vs 0.59, *P* = 0.046) than those without diaphragm paralysis. The use of an autologous pericardial patch in stage 1 was a risk factor for diaphragm paralysis development (odds ratio: 2.61, *P* = 0.012). Diaphragm paralysis was associated with an increased risk of protein-losing enteropathy (hazard ratio: 2.31, *P* = 0.003), particularly in patients without recovery after plication (hazard ratio: 4.85, *P* = 0.031).

**CONCLUSIONS:**

Diaphragm paralysis following Fontan completion significantly increases the risk of protein-losing enteropathy and long-term mortality, particularly in patients who fail to recover after plication. Early identification and appropriate management of diaphragm paralysis may be crucial for optimizing outcomes.

## Graphical abstract

**Figure:**
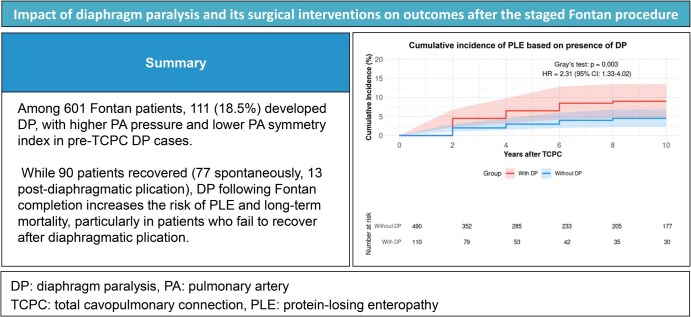

## INTRODUCTION

Diaphragm paralysis (DP), owing to phrenic nerve injury, is a well-known complication after congenital heart surgery [1–4]. Especially in neonates and young infants, phrenic nerve injury prolongs the duration of mechanical ventilation and is a source of subsequent morbidities [5, 6]. Additionally, paradoxical movement of the paretic diaphragm can compromise the function of the contralateral hemithorax. Respiratory dynamics are of particular relevance in children with single ventricle (SV) defects because pulmonary blood flow is influenced by changes in the intrathoracic pressure. The risk of DP might increase in children with SV defects through multiple surgical procedures due to the current strategy of staged Fontan palliation and to the increase of the more complex cases reaching Fontan circulation [7–9]. Although recovery of diaphragmatic function could be expected, diaphragm plication is needed in selected patients [10–12]. It is difficult to predict which patients will recover from DP and to which extent the function will recover. Kumar *et al.* [10] demonstrated that diaphragm plication does not adversely affect surgical outcomes at subsequent stages of SV palliation but does eliminate the variability associated with recovery of diaphragmatic function such that all patients will have favourable pulmonary haemodynamics and progress to Fontan completion. Hsia *et al.* [13] investigated subdiaphragmatic venous haemodynamics in patients with Fontan circulation after diaphragm plication and found that diaphragm plication does not completely restore normal subdiaphragmatic venous fluid dynamics, and inspiration-derived hepatic venous flow is suppressed. This suboptimal pulmonary and splanchnic circulation may contribute to early problems of prolonged pleural effusion and ascites and promote late Fontan failure [5, 6]. However, few studies evaluated the impact of DP, which occurred during the staged SV palliations, on late outcomes after the Fontan completion.

In this study, we sought to determine the incidence of DP in patients who underwent the staged Fontan procedure, to identify the risks for the development of DP, and to determine the impact of DP on outcomes following the Fontan procedure. We also evaluated the effects of diaphragm plication on the hospital course and the late outcomes after the Fontan procedure.

## METHODS

### Ethical statement

This study was approved by the Institutional Review Board of the Technical University of Munich (approval number 2024-334-S-CB on 8 July 2024). Because of the retrospective nature of the study, the need for individual patient consent was waived.

### Patients and data collection

This study reviewed all patients who underwent a staged Fontan procedure using a bidirectional cavopulmonary shunt (BCPS) and total cavopulmonary connection (TCPC) from 1994 to 2023. Patients with conversion from classic Fontan procedure to TCPC were excluded. Patient baseline and follow-up information was obtained from our SV database, which includes the initial stage I palliation (the Norwood procedure, aortopulmonary shunt, and pulmonary artery (PA) banding), BCPS and the TCPC. The follow-up period for each patient was defined as the interval between TCPC and the most recent clinical examination. Listwise deletion was used for missing data. Complete case analysis was used for the primary outcomes, as these had minimal missing data (<5%). Active protein-losing enteropathy (PLE) was defined by clinical symptoms (persistent diarrhoea, oedema, pleural effusions or ascites) with serum albumin <3.5 g/dl and total protein <6.0 g/dl, confirmed by elevated faecal alpha-1 antitrypsin levels [14]. PA symmetry index was calculated as the ratio of smaller to larger PA cross-sectional area measured immediately proximal to the first lobar branch on selective pulmonary angiography.

### Diagnosis of diaphragm paralysis

All patients underwent pre-BCPS/TCPC cardiac catheterization. When DP was suspected based on clinical findings (ventilator dependency, elevated diaphragm on X-ray, respiratory distress) and ultrasound, fluoroscopic evaluation was performed during catheterization. Normal diaphragm function (DF) was defined as symmetrical movement without paradox during spontaneous breathing. Recovery meant a return to normal symmetrical movement without paradox or significant contralateral elevation on fluoroscopy. Diaphragmatic plication was indicated for ventilator weaning failure or continuous nasal continuous positive airway pressure requirement post-extubation.

### Surgical techniques

The techniques for BCPS and TCPC were previously described [15–17]. Fenestration was not routinely performed, but only in high-risk patients, such as those with single-lung Fontan or reduced ventricular function [16]. Diaphragmatic plication was performed through a lateral sternotomy from the sixth or seventh intercostal space on the affected side, and a ‘central pleating’ technique was performed, which uses horizontal mattress sutures with Teflon pledgets in a line running lateral to medial on the hemidiaphragm to create folds and flatten the diaphragm by folding it into the centre [18, 19]. When diaphragmatic plications were performed concomitantly to BCPS or TCPC, they were accomplished through a median sternotomy.

### Statistical analysis

Categorical variables are presented as numbers and percentages, and continuous variables as medians with interquartile ranges (IQRs). Comparisons were made between patients who developed DP before TCPC and those who did not. Chi-squared tests were used for categorical variables. For continuous variables, Student’s *t*-tests were employed for normally distributed data and Mann–Whitney *U*-tests for non-normally distributed data, as determined by Levene’s test. Variables with *P* < 0.1 in univariable analysis and <10% missing data were included in multivariable logistic regression models to identify risk factors. Competing risk analyses were performed using the Aalen–Johansen estimator to calculate cumulative incidence rates, with death as a competing risk. Group comparisons were performed using Gray’s test, and hazard ratios (HRs) were estimated using Fine-Gray competing risk regression models. The following analyses were performed: (i) DP impact assessment, (ii) plication effect evaluation in DP, (iii) DF recovery outcome analysis, and (iv) paralysis onset timing examination across three stages—pre-BCPS (stage 1), BCPS-to-TCPC interval (stage 2), and at TCPC (stage 3). Cox proportional hazard models were used to identify risk factors for PLE, with variables significant in univariable analyses (*P* < 0.05) included in the multivariable model. Proportional hazard assumptions were verified using Schoenfeld residuals. ALL analyses were performed with the Statistical Package for the Social Sciences version 28.0 for Windows (IBM, Ehningen, Germany) and R-statistical software (R Foundation for Statistical Computing, Vienna, Austria).

## RESULTS

### Patient demographics and clinical characteristics

Among the 601 patients who underwent staged Fontan completion, 111 patients (18.5%) developed DP, with 79 patients (13.1%) developing DP before TCPC and 32 patients (5.3%) after TCPC. The median follow-up was 4.6 years (IQR: 1.7–12.2) in the DP group and 5.5 years (IQR: 1.6–13.7) in the no DP group. Patient characteristics with and without DP before TCPC are shown in Table 1. Patients with pre-TCPC DP showed higher pulmonary arterial pressure (PAP, 10 (IQR: 8–11) vs 9 (IQR: 7–11) mmHg, *P* = 0.045) and lower PA symmetry index (0.54 (IQR: 0.30–0.71) vs 0.59 (IQR: 0.39–0.77), *P* = 0.046) compared to those without (Table 2). Perioperative and hospital outcomes are similar between the groups (Supplementary Table S1).

**Table 1:** Baseline cohort characteristics

Variables: *N* (%)	Total	DP	No DP	
		Before TCPC	Before TCPC	
Number of patients	601	79 (13.1)	522 (86.9)	
Male sex	370 (61.6)	51 (64.6)	319 (61.1)	
Primary diagnosis				
HLHS	175 (29.1)	28 (35.4)	147 (28.2)	
Tricuspid atresia	97 (16.1)	9 (11.4)	88 (16.9)	
DILV	81 (13.5)	8 (10.1)	73 (14.0)	
PA/IVS	32 (5.3)	4 (5.1)	28 (5.4)	
UAVSD	25 (4.2)	1 (1.3)	24 (4.6)	
Single ventricle	121 (20.1)	16 (20.3)	105 (20.1)	
Associated anomaly				
TGA	185 (30.8)	22 (27.8)	163 (31.2)	
DORV	76 (12.6)	10 (12.7)	66 (12.6)	
CoA	76 (12.6)	6 (7.6)	70 (13.4)	
ccTGA	27 (4.5)	3 (3.8)	24 (4.6)	
Dextrocardia	54 (9.0)	5(6.3)	49 (9.4)	
Heterotaxy	45 (7.5)	5 (6.3)	40 (7.7)	
TAPVC	38 (6.3)	5 (6.3)	33 (6.3)	
Dominant right ventricle	323 (53.7)	50 (63.3)	273 (52.3)	
Baseline characteristics				
Norwood as first palliation	282 (46.9)	41 (51.9)	241 (46.2)	
Using auto-pericardium at Norwood	41 (6.8)	13 (16.5)	28 (5.4)	
Number of palliations ≥3	57 (9.5)	9 (11.4)	48 (9.2)	

HLHS: hypoplastic left heart syndrome, DILV: double inlet left ventricle, PA/IVS: pulmonary atresia and intact ventricular septum, UAVSD: unbalanced atrioventricular septum, TGA: transposition of the great arteries, DORV: double outlet right ventricle, CoA: coarctation of the aorta, ccTGA: congenitally corrected transposition of the great arteries, TAPVC: total anomalous pulmonary venous connection.

**Table 2:** Mean differences in cardiac catheterization between patients with and without diaphragm paralysis

Variables: mean	DP(+) before TCPC	DP(−) before TCPC	Mean difference (95% CI)	*P*-value
Preoperative data before TCPC				
PAP	10.04	9.37	0.67 (0.01, 1.32)	0.045
TPG	3.82	3.70	0.12 (−0.30, 0.55)	0.571
LAP	5.84	5.58	0.26 (−0.34, 0.85)	0.394
SVP	85.50	84.12	1.38 (−1.78, 4.53)	0.391
SVEDP	7.70	7.77	−0.07 (−0.75, 0.62)	0.848
SO_2_	83.25	82.25	1.00 (−0.60, 2.60)	0.220
PA index	178.22	179.67	−1.46 (−21.41, 18.50)	0.886
PA symmetry index	0.52	0.59	−0.07 (−0.14, −0.001)	0.046

DP: diaphragm paralysis, TCPC: total cavopulmonary connection, PAP: pulmonary artery pressure, TPG: transpulmonary gradient, LAP: left atrial pressure, SVP: systolic ventricular pressure, SVEDV: systemic ventricular end diastolic pressure, SO2: arterial oxygen saturation, PA: pulmonary artery.

### Recovery patterns and risk factors

Among DP patients, 77 had a spontaneous recovery, 13 recovered after plication and 21 remained without recovery (Table 3). As shown in Supplementary Table S2, logistic regression analysis identified autologous pericardial patch use in stage 1 as a significant risk factor for DP (*P* = 0.012, odds ratio = 2.61). Multivariable analysis identified two independent risk factors for PLE: diaphragmatic plication (adjusted HR: 4.02, 95% confidence interval (CI): 1.38–11.67, *P* = 0.011) and elevated pre-TCPC PAP (adjusted HR: 1.23 per mmHg increase, 95% CI: 1.11–1.35, *P* < 0.001, Supplementary Table S3).

**Table 3:** Distribution of diaphragm paralysis following staged Fontan operation

Variables: *N*	Group	Total DP	Recovered DM		Non-recoverd DM	
			Plication(+)	Plication(−)	Plication(+)	Plication(−)
Number of patients		111	13	77	9	12
Post-stage 1 palliation	DP before TCPC	33	4	22	6	1
Post-BCPS		46	7	33	2	4
Post-TCPC	DP after TCPC	32	2	22	1	7

### Protein-losing enteropathy risk and long-term outcomes

**Figure 1:**
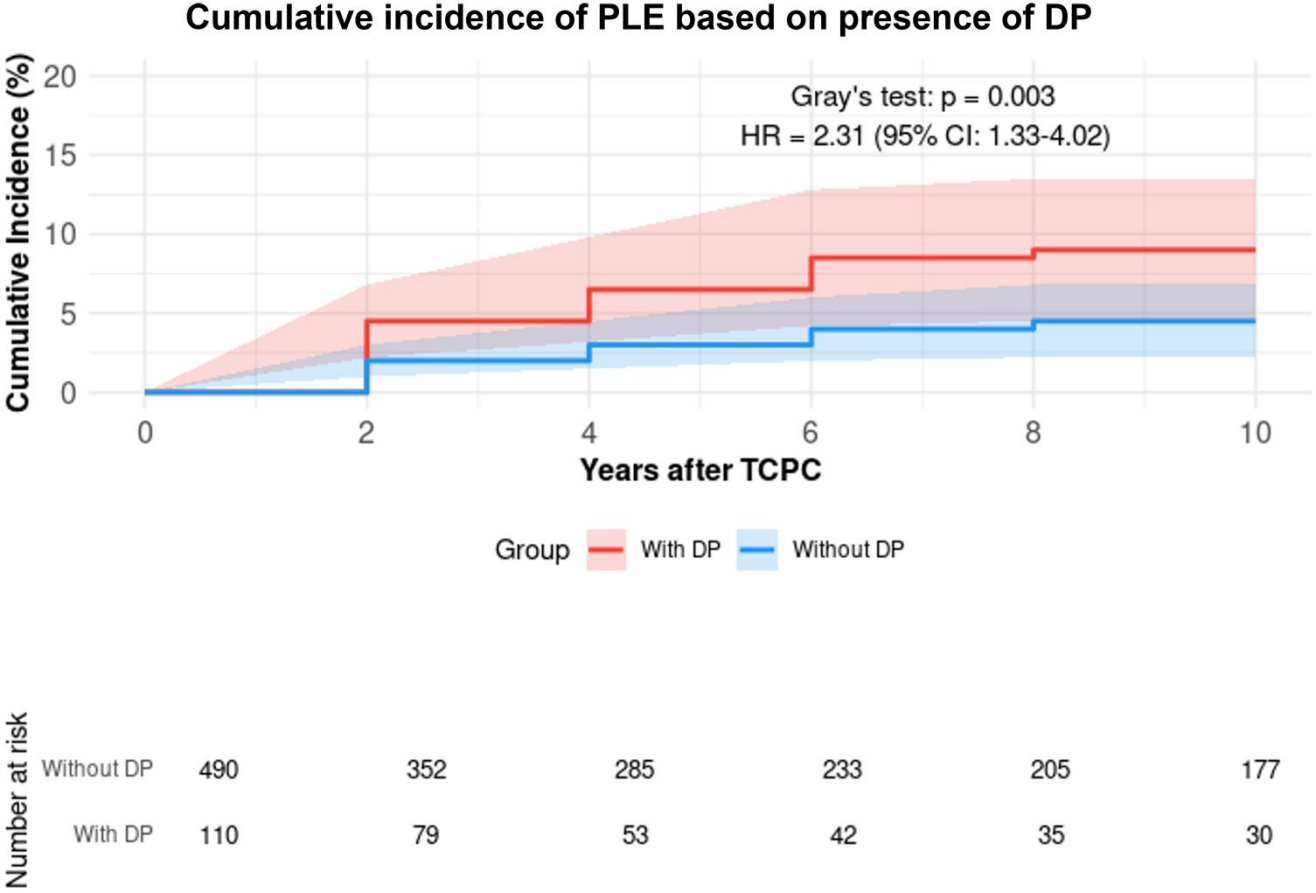
Cumulative incidence of PLE based on presence of DP

**Figure 2:**
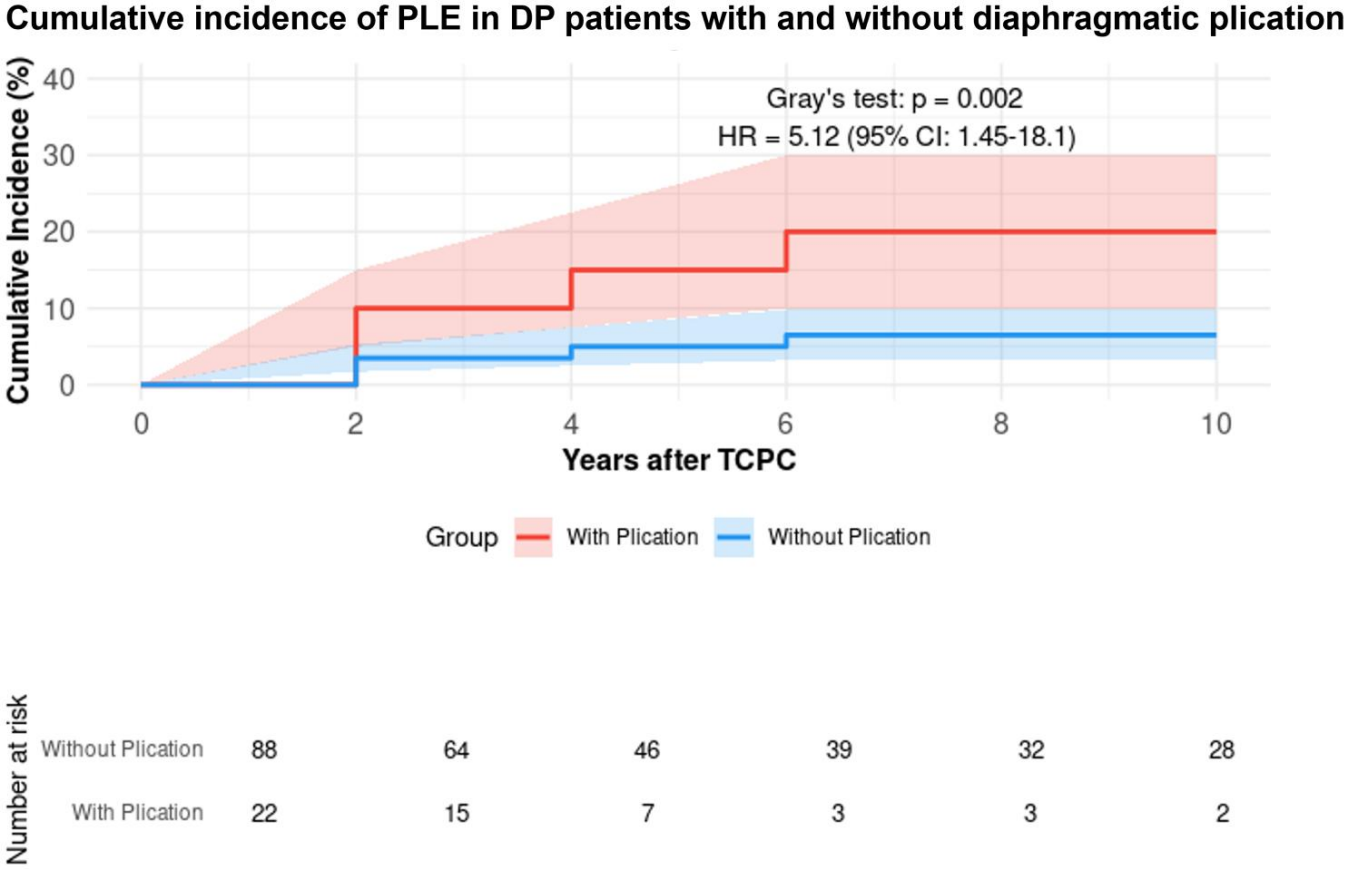
Cumulative incidence of PLE in DP patients with and without diaphragmatic plication

**Figure 3:**
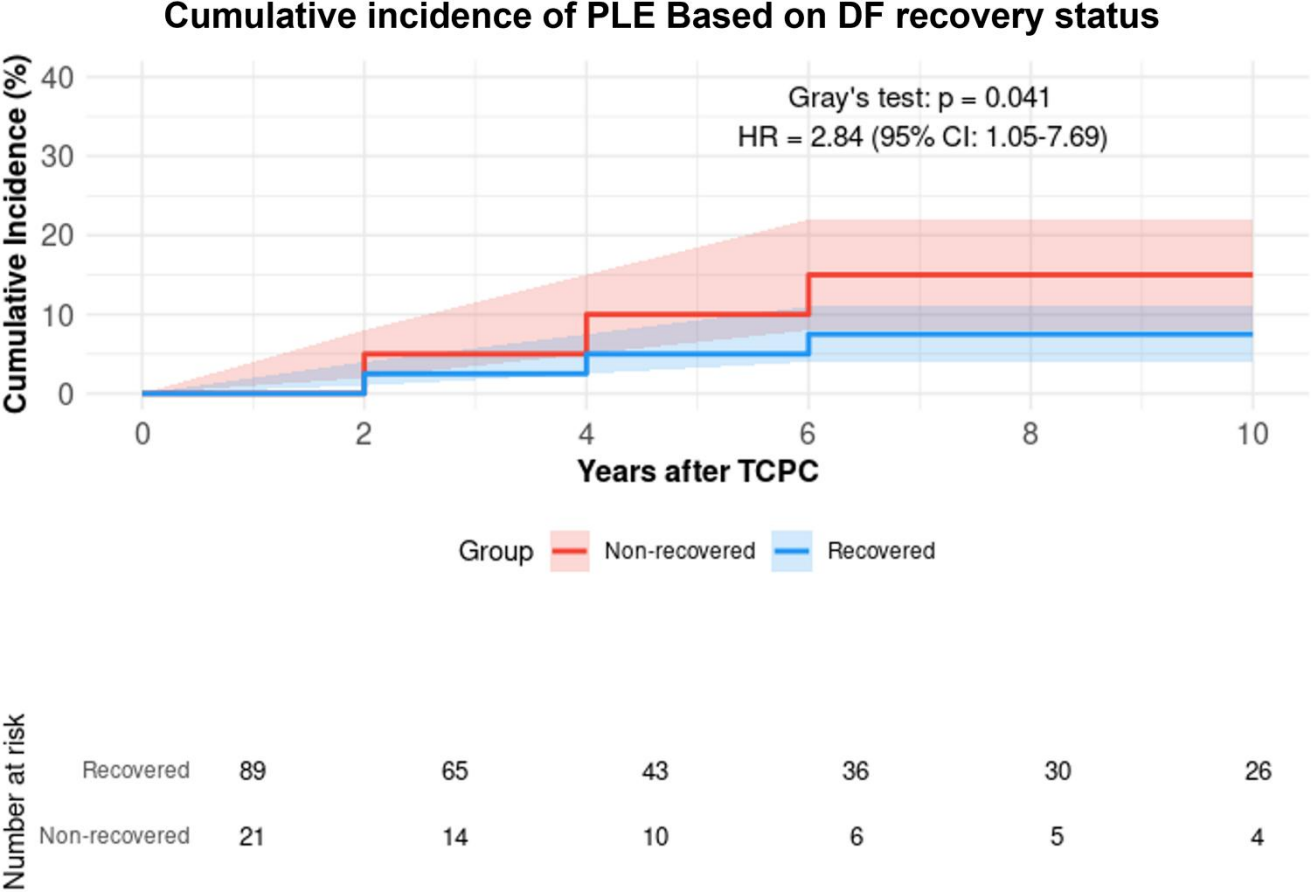
Cumulative incidence of PLE based on DF recovery status

**Figure 4:**
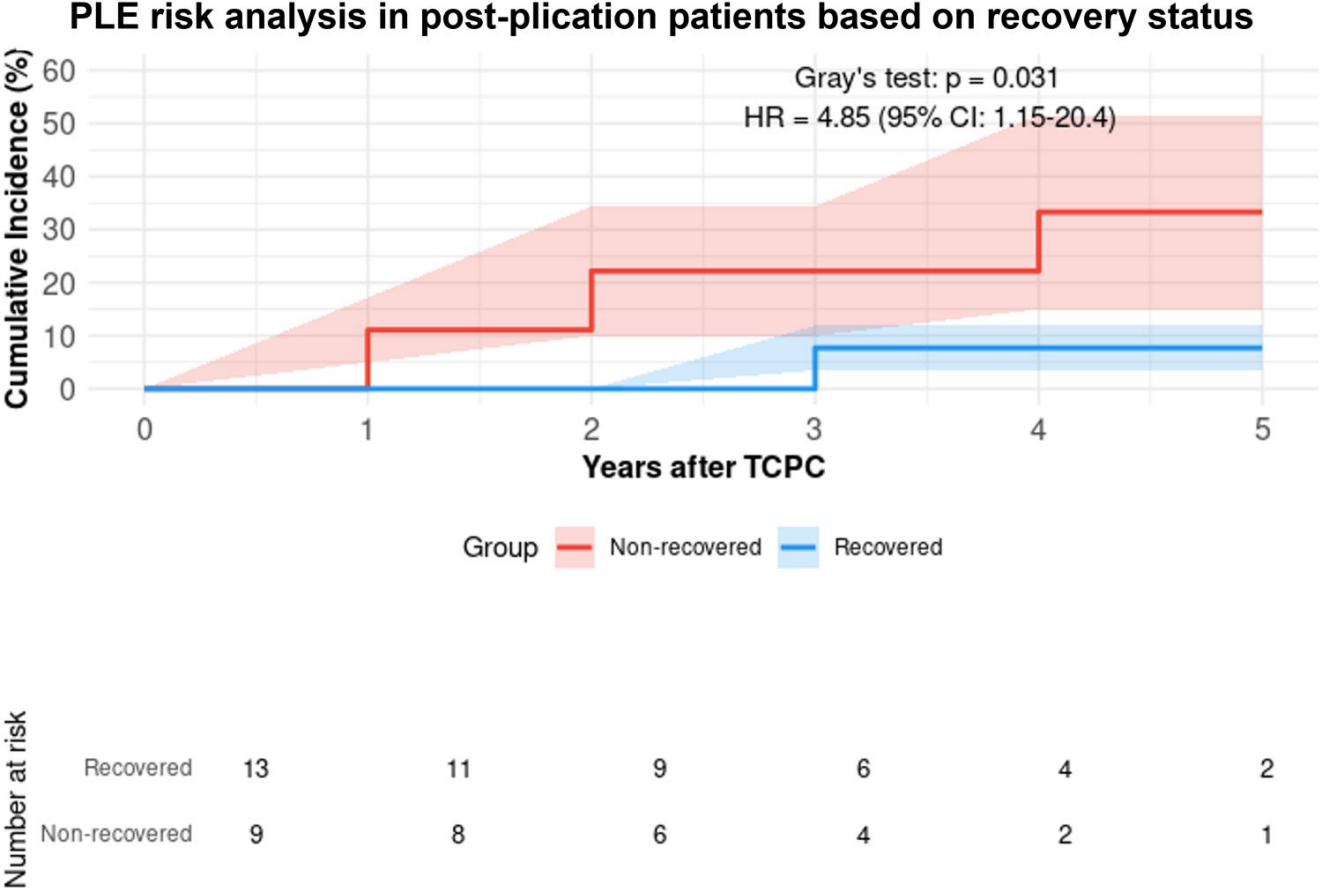
PLE risk analysis in post-diaphragmatic plication patients based on recovery status

Patients with DP showed a higher risk of PLE compared to those without (*n* = 490, 81.5%) (HR: 2.31, 95% CI: 1.33–4.02, *P* = 0.003), with 10-year cumulative incidence rates of 9.0% and 4.5%, respectively (Fig. 1). Among paralysis patients, those who underwent diaphragmatic plication (*n* = 19) demonstrated a significantly higher PLE risk compared to those without diaphragmatic plication (*n* = 60) (HR: 5.12, 95% CI: 1.45–18.1, *P* = 0.002, Fig. 2). Diaphragm recovery status significantly influenced outcomes, with non-recovery patients (*n* = 21) showing higher PLE risk compared to those with recovery (*n* = 90) (HR: 2.84, 95% CI: 1.05–7.69, *P* = 0.041, Fig. 3). This effect was particularly pronounced in patients who underwent diaphragmatic plication (*n* = 22), where non-recovery was associated with a markedly higher risk (HR: 4.85, 95% CI: 1.15–20.4, *P* = 0.031, Fig. 4). The timing of paralysis onset showed no significant impact on PLE development (*P* = 0.892) across stage 1 (*n* = 33), stage 2 (*n* = 46) and stage 3 (*n* = 32) groups (Supplementary Fig. S1). At 20 years, while heart transplantation rates were similar between groups (DP: 3.8% (95% CI: 0.0–11.4%) versus no DP: 2.5% (95% CI: 0.0–6.1%), *P* > 0.05), mortality was higher in the DP group (32.1% (95% CI: 11.3–52.9%) vs 2.4% (95% CI: 0.4–4.4%)).

## DISCUSSION

Among the 601 Fontan patients, 111 (18.5%) developed DP, with higher PAP and lower PA symmetry index in pre-TCPC DP cases. While 90 patients recovered (77 spontaneously, 13 post-plication), DP was associated with increased PLE risk (HR: 2.31, *P* = 0.003), particularly in non-recovery cases post-plication (HR: 4.85, *P* = 0.031).

### Incidence of diaphragm function

The phrenic nerve is susceptible to damage, especially in small infants, due to possible lesions caused by preparation, especially during repeat surgeries, mechanical strain, contusion and impact of hypothermia. Previous studies have reported the incidence of DP after congenital heart surgery of 1–16% [1–6, 20, 21]. The incidence of DP might vary according to the type of procedure and also to the age at operation. Akay *et al.* [1] reported a high incidence of DP following tetralogy of Fallot repair, Blalock Taussig Tomas shunt and ventricular septal defect (VSD) closure. Talwar *et al.* [3] demonstrated that the incidence of DP is particularly high after the BCPS, Fontan operation, Blalock Taussig Tomas shunt, VSD closure, tetralogy of Fallot repair and arterial switch operation. Parmar *et al.* [21] reported a high incidence of DP after arterial switch operation, total anomalous pulmonary venous connection repair, VSD closure, aortic arch repair and Blalock Taussig Tomas shunt. DP during SV palliation should be cumulatively higher because patients usually need multiple palliations. Previous studies demonstrated a 3–13% incidence of DP after the Fontan procedure [7–9]. Our results of 13% before TCPC and 5% following TCPC were consistent with these reports. In the present study, we observed a higher incidence of DP in patients where the pericardium was harvested for aortic arch repair. Resection of the pericardium and the sequelae of adhesions and their dissection during subsequent operations close to the phrenic nerve may increase the risk of phrenic nerve injury.

### Impact of diaphragm paralysis on respiratory dynamics and haemodynamics

The negative impact of DP on respiratory dynamics and pulmonary circulation has been well described [1–7, 22]. Respiratory dynamics are of particular importance in children with SV physiology, given that pulmonary blood flow is influenced by changes in the intrathoracic pressure. Hsia *et al.* reported alterations in subdiaphragmatic venous haemodynamics in Fontan patients after diaphragm plication. They postulated that these changes in venous pressures might cause prolonged pleural effusions and potentially contribute to late Fontan failure [22]. Although our results demonstrated higher PAP and lower PA symmetric index before TCPC, no adverse impact of DP on the postoperative course was observed.

### Recovery of diaphragm paralysis

It has been shown that DP after paediatric cardiac surgery is not permanent in all patients. Given our practice of routine catheterization before the second and third stage palliation, we can demonstrate the recovery of diaphragmatic function. The findings in the previous studies and our present study confirm that two-thirds of patients demonstrate recovery of diaphragmatic function. However, one-quarter of those who did not recover from DP demonstrated poor outcomes. While our data showed no significant impact of DP timing on PLE development, the use of an autologous pericardial patch in stage 1 was identified as a risk factor for DP, suggesting potential surgical technique-related factors in early stages may influence long-term outcomes.

### Indication for and effects of diaphragm plication

Previous studies have shown that hemidiaphragm plication improves short-term outcomes [2, 11–13]. However, little is known about the effect of plication on the outcomes of subsequent staged Fontan palliation. Recently, Kumar *et al.* [10] reported 30 patients with SV who underwent diaphragm plication for DP before Fontan completion (19 after stage I and 11 after BCPS) and showed that no difference was found in PAP, pulmonary vascular resistance or postoperative course between the plicated patients and their propensity-matched controls at the time of the Fontan completion. They concluded that their results suggest an aggressive indication of plication in patients with DP. The important issue is the recovery of diagrammatic function at the time of TCPC. Although we could not demonstrate the significant effects of diaphragm plication on late outcomes, we agree with an aggressive indication of plication in patients with DP. Thus, early fluoroscopy is pursued in these patients to confirm or rule out the suspicion.

### Impact of diaphragm paralysis on outcomes after total cavopulmonary connection

Previous studies have shown that DP increases in-hospital morbidity after staged Fontan palliations, such as prolonged mechanical ventilation, prolonged pleural effusions, prolonged hospital stay and increased use of antibiotics [6–8, 23–25]. However, few studies demonstrated the impact of DP on long-term outcomes after the Fontan procedure. Ovroutski *et al.* [7] showed that patients with DP developed chronic ascites more frequently than those without DP during the median follow-up of 4.6 years postoperatively. Lemmer *et al.* [11] demonstrated that exercise capacity in patients with DP was inferior to those without after the Fontan procedure. To the best of our knowledge, our study is the first to report the impact of DP on the development of PLE. We suspect that DP causes impaired lymph flow, similar to those observed in patients with failing Fontan circulation. This suboptimal splanchnic circulation may contribute to promoting PLE.

### Study limitations

This study had a retrospective, single-centre design, with final haemodynamic assessment by cardiac catheterization limited to pre-TCPC data. A major limitation is the non-standardized documentation of diaphragm motion patterns (paradoxical versus immobile non-paradoxical) over the 30-year study period. A prospective study with standardized post-TCPC catheterization protocols and diaphragm motion assessment would better clarify the long-term impact of DP and determine whether plication is more beneficial for certain types of dysfunction in preventing late complications, including PLE.

## CONCLUSIONS

DP following Fontan completion increases the risk of PLE and long-term mortality, particularly in patients who fail to recover after plication. While the timing of paralysis onset did not affect outcomes, careful patient selection for diaphragmatic plication and vigilant monitoring of DF are crucial for optimizing long-term results.

## Supplementary Material

ivaf072_Supplementary_Data

## Data Availability

The data underlying this article will be shared by the corresponding author on reasonable request.

## Notes

Presented at the 38th Annual Meeting of the European Association for Cardio-Thoracic Surgery, Lisbon, Portugal, October 9–12, 2024.

## ABBREVIATIONS

BCPS: Bidirectional cavopulmonary shunt
CI: Confidence interval
DF: Diaphragm function
DP: Diaphragm paralysis
HR: Hazard ratio
IQR: Interquartile ranges
PA: Pulmonary artery
PAP: Pulmonary artery pressure
PLE: Protein-losing enteropathy
TCPC: Total cavopulmonary connection
SV: Single ventricle
VSD: Ventricular septal defect
