# Supplementary material for: Impact of diaphragm paralysis and its surgical interventions on outcomes after the staged Fontan procedure
Source: Interdiscip Cardiovasc Thorac Surg. 2025 Mar 19;40(4):ivaf072. doi: 10.1093/icvts/ivaf072 (PMC11955237; doi:10.1093/icvts/ivaf072)
Supplement: ivaf072_Supplementary_Data [file ivaf072_supplementary_data.zip › CORRECT Supplementary Table.docx]

**Supplementary Table**

**Supplementary Table S1:**

| Supplementary Table S1: Perioperative variables in TCPC | | | | |
| --- | --- | --- | --- | --- |
| **Variables: N(%) or median (IQR)** | **Total** | **DP** | **No DP** | **p-value** |
|  |  | **before TCPC** | **before TCPC** |  |
| Number of patients | 601 | 79 | 522 |  |
| **Operative data** | | | | |
| Age at TCPC (years) | 2.2 (1.8-2.9) | 2.3 (1.8-2.9) | 2.2 (1.8-2.9) | 0.745 |
| Weight at TCPC (kg) | 11.7 (10.6-13.5) | 11.9 (10.8-13.5) | 11.6 (10.6-13.5) | 0.591 |
| CPB time (min) | 64 (47-93) | 63 (43-86) | 64 (47-93) | 0.627 |
| Extracardiac TCPC | 576 (95.8) | 76 (96.2) | 500 (95.8) | 0.863 |
| Fenestrated TCPC | 42 (7.0) | 6 (7.6) | 36 (6.9) | 0.820 |
| **Post-TCPC hospital data** | | | | |
| Duration of MV (hrs) | 5 (3-11) | 5 (3-10) | 5 (3-11) | 0.542 |
| Prolonged effusion > 14 days | 62 (10.3) | 7 (8.9) | 55 (10.5) | 0.743 |
| Chylothorax | 134 (22.3) | 19 (24.1) | 115 (22.0) | 0.714 |
| Ascites | 117 (19.5) | 19 (24.1) | 98 (18.8) | 0.277 |
| Secondary Fenestration needed | 11 (1.8) | 2 (2.5) | 9 (1.7) | 0.618 |
| ICU stay (days) | 6 (4-8) | 6 (4-9) | 6 (4-8) | 0.650 |
| Hospital stay (days) | 20 (14-26) | 21 (15-31) | 19 (14-26) | 0.123 |
| Hospital stay <14 days | 179 (29.8) | 18 (22.8) | 161 (30.8) | 0.140 |
| Re-ICU admission | 149 (24.8) | 19 (24.1) | 130 (24.9) | 0.849 |

**Supplementary Table S2:**

| Supplementary Table S2: Risk factors for diaphragm paralysis | | | | | | | |
| --- | --- | --- | --- | --- | --- | --- | --- |
| **Variables** | Univariate | | |  | Multivariate | | |
|  | Odds Ratio | 95% CI | p-value |  | Odds Ratio | 95% CI | p-value |
| **Primary Diagnosis** | | | | | | | |
| HLHS | 1.28 | 0.82-1.98 | 0.280 |  |  |  |  |
| Tricuspid atresia | 0.58 | 0.30-1.10 | 0.094 |  |  |  |  |
| DILV | 1.10 | 0.61-1.99 | 0.749 |  |  |  |  |
| PA/IVS | 0.62 | 0.21-1.80 | 0.375 |  |  |  |  |
| UAVSD | 0.59 | 0.17-2.01 | 0.400 |  |  |  |  |
| Single ventricle | 0.91 | 0.54-1.54 | 0.724 |  |  |  |  |
| **Associated anomaly** | | | | | | | |
| TGA | 0.85 | 0.54-1.33 | 0.471 |  |  |  |  |
| DORV | 1.00 | .054-1.85 | 0.991 |  |  |  |  |
| CoA | 0.72 | 0.37-1.41 | 0.339 |  |  |  |  |
| ccTGA | 1.28 | 0.50-3.24 | 0.608 |  |  |  |  |
| Dextrocardia | 1.00 | 0.49-2.06 | 0.992 |  |  |  |  |
| Heterotaxy | 0.95 | 0.43-2.10 | 0.901 |  |  |  |  |
| TAPVC | 0.65 | 0.25-1.71 | 0.387 |  |  |  |  |
| Dominant right ventricle | 1.27 | 0.84-1.93 | 0.260 |  |  | | |
| **Baseline characteristics** | | | | | | | |
| DKS/NW at 1st palliation | 1.30 | 0.86-1.96 | 0.213 |  |  |  |  |
| Using auto-pericardium at DKS/NW | 3.13 | 1.61-6.09 | <0.001 |  | 2.73 | 1.36-5.47 | 0.005 |
| No of Palliation ≥ 3 | 2.31 | 1.10-3.74 | 0.022 |  |  |  |  |

**Supplementary Table S3:**

| Supplementary Table S3: Risk factors for PLE | | | | | | | |
| --- | --- | --- | --- | --- | --- | --- | --- |
|  | **Univariable** | | |  | **Multivariable** | | |
| **Risk factor** | HR | 95% CI | p value |  | HR | 95% CI | p value |
| **Primary Diagnosis** | | | | | | | |
| HLHS | 1.588 | 0.769-3.278 | 0.211 |  |  |  |  |
| Dominant right ventricle | 2.277 | 1.052-4.927 | 0.037 |  |  |  |  |
| **Baseline characteristics** |  |  |  |  |  |  |  |
| Using auto-pericardium at stage I | 0.577 | 0.079-4.241 | 0.589 |  |  |  |  |
| No of Palliation ≥ 3 | 1.790 | 0.540-5.939 | 0.341 |  |  |  |  |
| **Preoperative data before TCPC** | | | | | | | |
| PAP | 1.244 | 1.131-1.368 | <0.001 |  | 1.228 | 1.114-1.354 | <0.001 |
| PA symmetry index | 0.484 | 0046-5.087 | 0.545 |  |  |  |  |
| **Diaphragm paralysis** | | | | | | | |
| DP after stage I | 1.802 | 0.548-5.932 | 0.332 |  |  |  |  |
| DP after BCPS | 1.977 | 0.692-5.653 | 0.203 |  |  |  |  |
| DP after TCPC | 2.065 | 0.628-6.789 | 0.232 |  |  |  |  |
| Diaphragmc plication | 5.368 | 1.855-15.539 | 0.002 |  | 4.016 | 1.383-11.666 | 0.011 |
