# Supplementary figures and images for: Impact of diaphragm paralysis and its surgical interventions on outcomes after the staged Fontan procedure
Source: Interdiscip Cardiovasc Thorac Surg. 2025 Mar 19;40(4):ivaf072. doi: 10.1093/icvts/ivaf072 (PMC11955237; doi:10.1093/icvts/ivaf072)

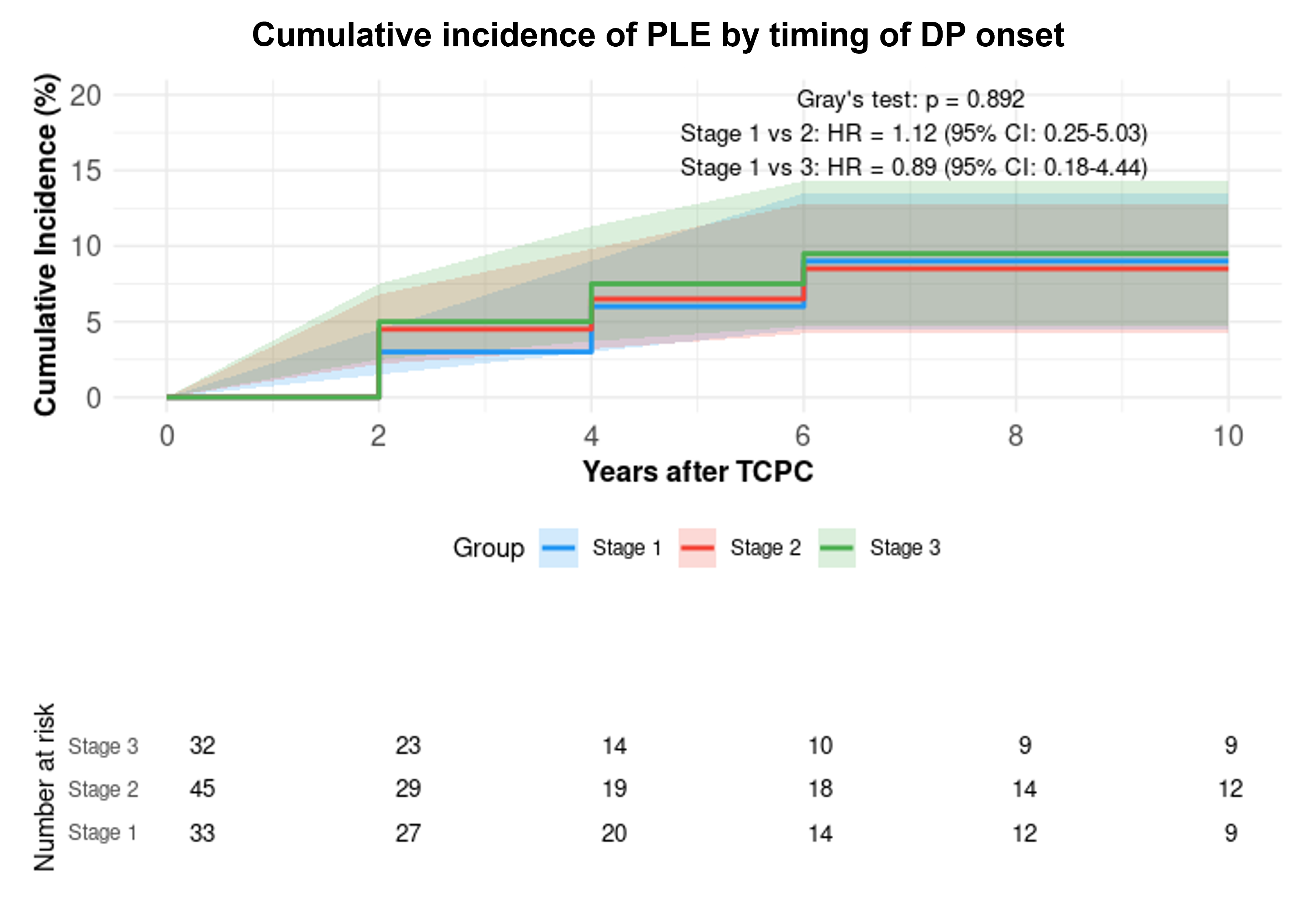

Supplement: ivaf072_Supplementary_Data [file ivaf072_supplementary_data.zip › Supplementary Figure 01.png]
